# Liquid biopsy techniques and pancreatic cancer: diagnosis, monitoring, and evaluation

**DOI:** 10.1186/s12943-023-01870-3

**Published:** 2023-10-06

**Authors:** Kangchun Wang, Xin Wang, Qi Pan, Bei Zhao

**Affiliations:** 1 Department of Organ Transplantation and Hepatobiliary, The First Affiliated Hospital of China Medical University, Shenyang, 110001 China; 2 Movement System Injury and Repair Research Center, Xiangya Hospital, Central South University, Changsha, 410008 China; 3 Department of Ultrasound, The Third Affiliated Hospital of Guangzhou Medical University, Guangzhou, 510150 China

**Keywords:** Circulating tumour cells, Circulating tumour DNA, Noncoding RNAs, Extracellular vesicles, Exosomes, Pancreatic cancer, Liquid biopsy

## Abstract

Pancreatic cancer (PC) is one of the most common malignancies. Surgical resection is a potential curative approach for PC, but most patients are unsuitable for operations when at the time of diagnosis. Even with surgery, some patients may still experience tumour metastasis during the operation or shortly after surgery, as precise prognosis evaluation is not always possible. If patients miss the opportunity for surgery and resort to chemotherapy, they may face the challenging issue of chemotherapy resistance. In recent years, liquid biopsy has shown promising prospects in disease diagnosis, treatment monitoring, and prognosis assessment. As a noninvasive detection method, liquid biopsy offers advantages over traditional diagnostic procedures, such as tissue biopsy, in terms of both cost-effectiveness and convenience. The information provided by liquid biopsy helps clinical practitioners understand the molecular mechanisms underlying tumour occurrence and development, enabling the formulation of more precise and personalized treatment decisions for each patient. This review introduces molecular biomarkers and detection methods in liquid biopsy for PC, including circulating tumour cells (CTCs), circulating tumour DNA (ctDNA), noncoding RNAs (ncRNAs), and extracellular vesicles (EVs) or exosomes. Additionally, we summarize the applications of liquid biopsy in the early diagnosis, treatment response, resistance assessment, and prognostic evaluation of PC.

## Introduction

Pancreatic cancer (PC) is one of the most common malignancies, and the number of PC cases has doubled over the past two decades. The incidence of PC varies significantly across regions and populations, with the highest rates observed in North America, Europe, and Australia [1–3]. Recent years have seen a rapid increase in deaths due to PC, which can be attributed to global population growth and age structure changes and is closely linked to social and economic development [4]. According to predictions, PC is expected to become the third leading cause of cancer-related deaths in the European Union [5]. By 2030, it is projected to overtake breast, prostate, and colorectal cancers and become the second leading cause of cancer-related deaths in the United States [6].

Pancreatic ductal adenocarcinoma (PDAC) is a primary histological subtype of PC, accounting for 90% of all cases [7, 8]. Surgical resection is one of the methods of a potential cure, but most PDAC patients are unsuitable for operations when they are diagnosed [9, 10]. Therefore, screening and diagnosis should be conducted as early as possible to ensure a positive outcome. The diagnosis of PDAC relies on endoscopic ultrasonography-guided fine needle aspiration (EUS-FNA), magnetic resonance imaging (MRI), and computed tomography (CT) [11–14]. However, there are some problems, such as invasiveness, high cost, and exposure of subjects to radiation [15, 16]. In addition, the molecular composition of tumours is complex and dynamic, and repeated endoscopy examinations create a significant burden on patients [17]. Although the potential role of diagnostic biomarkers of cancer is constantly evolving, reliable diagnostic biomarkers for PC are still lacking. For instance, carbohydrate antigen 19-9 (CA19-9) has been extensively studied as a biomarker for detecting PC [18]. However, due to the lack of specificity of CA19-9, it can be expressed in various liver and gallbladder diseases as well as other types of malignant tumours, and elevated levels can also occur in some benign obstructive diseases [19]. Fucosyltransferase 3 (also known as the Lewis gene) is the key enzyme involved in the biosynthesis of CA19-9. Approximately 5–10% of individuals are Lewis antigen-negative, which means they do not secrete or secrete very little CA19-9, and to some extent, this also hinders the diagnosis of PC [20]. Therefore, CA19-9 alone cannot offer a conclusive diagnosis and must be combined with different clinical presentations, imaging tests, and biomarkers [15, 21].

**Fig. 1:**
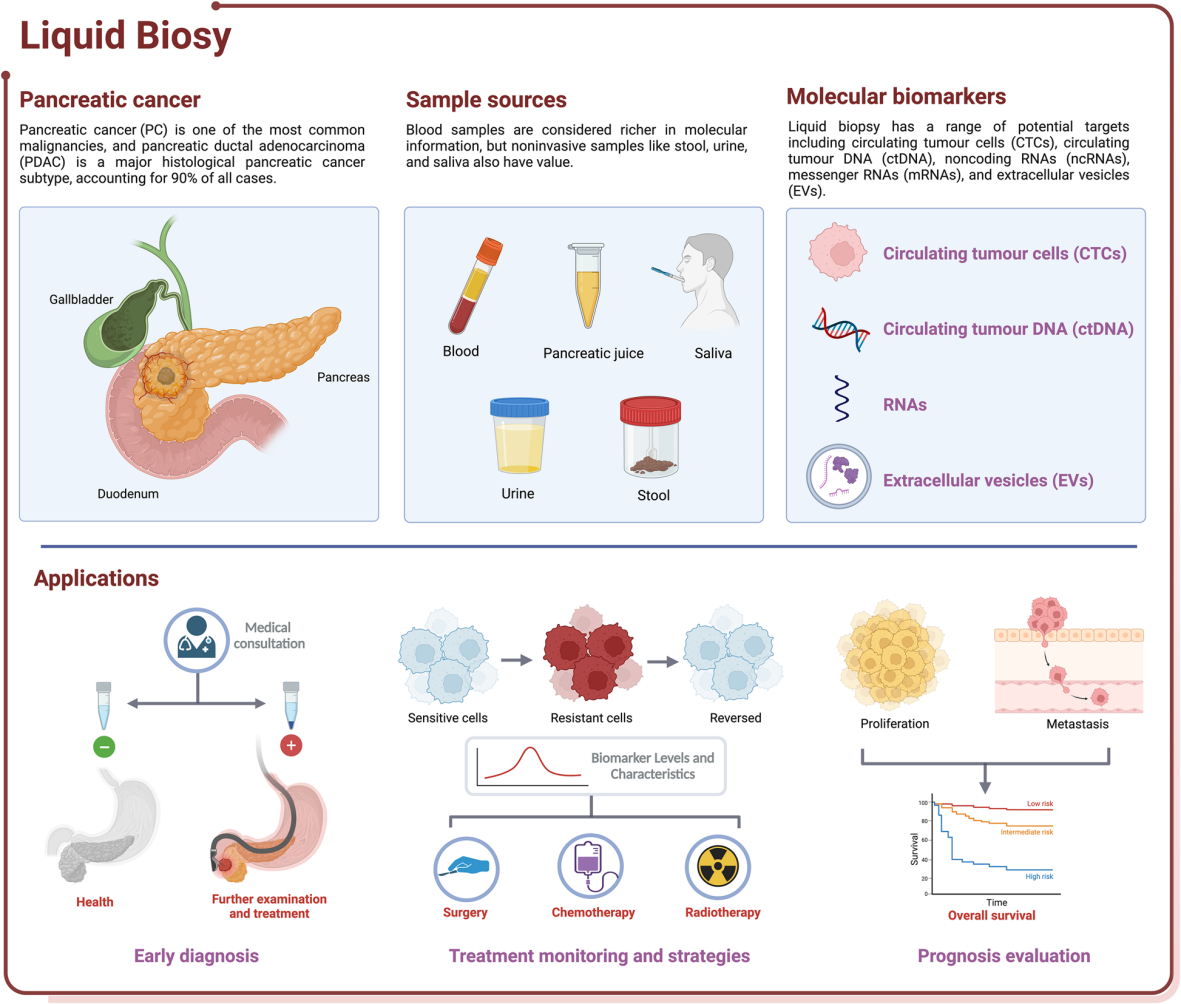
Common samples, biomarkers, and clinical applications in liquid biopsy for pancreatic cancer. Blood is typically the most commonly used material in liquid biopsy, in addition to pancreatic juice, saliva, urine, and stool. Circulating tumour cells, circulating tumour DNA, noncoding RNAs, and extracellular vesicles are among the most common biomarkers. Liquid biopsy has a wide range of clinical applications, playing a crucial role in early diagnosis, treatment monitoring, and prognosis evaluation. Created with BioRender.com

In recent years, liquid biopsy has garnered attention due to its advantages of lower invasiveness and the ability to continuously monitor cancer progression. While blood is considered the most critical biofluid for liquid biopsy (Fig. 1), other clinical samples, such as cerebrospinal fluid, saliva, ascites, pleural effusion, and urine, have also been used [22–26]. Different sample sources have unique characteristics, with the prevailing view suggesting that blood samples carry a richer molecular information profile. Although noninvasive samples such as stool, urine, and saliva may contain less biomarker information than blood, they can provide valuable information about the location of diseases. For instance, certain biomarkers in urine may be associated with kidney or bladder conditions [27], while stool biomarkers may be linked to digestive system disorders [28]. Currently, the potential targets of liquid biopsy are circulating tumour cells (CTCs), circulating tumour DNA (ctDNA), noncoding RNAs (ncRNAs), messenger RNAs (mRNAs), and extracellular vesicles (EVs), which can provide information about tumour genomics, transcriptomics, and proteomics.

Liquid biopsy exhibits high utility in the management of PC, with applications spanning early diagnosis, treatment strategies, drug resistance, recurrence monitoring, and prognosis assessment for PC patients. This review provides an overview of the biomarkers and detection methods utilized in liquid biopsy and their applications in the early diagnosis, treatment response, and prognosis evaluation of PC (Table 1). We also discuss the future trends of liquid biopsy and assess its limitations to improve current management strategies for patients.

**Table 1:** The isolation and detection techniques in liquid biopsy

	**Purpose**	**Methodology**	**Advantages**	**Disadvantages**	**Ref**
**CTCs**	isolation	density centrifugation	ease of operation	low detection rates of 24% to 40% in PDAC patients	[29]
		CTCs filters	isolation of CTCs without capture agents	small-sized CTCs might be overlooked	[30]
		flow cytometry	one of the most commonly used cell-sorting technologies for the analysis	lacked the ability to reveal sufficient morphological information to satisfy the standards set by pathologists for CTCs	[31]
		microfluidic devices	the CTCs chip captures large numbers of viable CTCs in a single step	complex manufacturing process and high cost	[32]
		dielectrophoresis	manipulate cells in accordance with their phenotype and membrane capacitance without the need for labeling or modification	Continuous optimization and fine-tuning of the electric field parameters are required	[33]
		immuno-magnetic separation	excellent speed and efficiency for CTCs detection and characterization	EpCAM-based strategy fails to detect CTCs with low EpCAM expression	[34]
	detection	high-resolution image scanning	an enrichment-free method to identify CTCs	limited applicability and processing speed	[35]
		mRNAs analysis and immunocytological staining	commonly used high-accuracy detection methods	complex operational procedures	[36]
		mutational analysis	provide information on tumour evolution	a single mutation cannot fully represent the overall characteristics	[37]
		single-cell next-generation sequencing	precise analysis of individual CTCs and revelation of tumour heterogeneity	complex sample handling	[38]
	combined strategy	integrated platform combined deterministic lateral displacement, inertial focusing and magnetophoresis	high throughput and efficiency, isolating CTCs regardless of tumour surface epitopes	high manufacturing costs and complexity	[39]
		negative selection and 3D cell culture	compared to traditional negative selection, it greatly improves purity	cell proliferation cultured in a 3D environment is slower	[40]
**CtDNA/ctRNA**	mutation detection	ddPCR	high sensitivity	requires a larger peripheral blood volume	[41]
		COLD-PCR	suitable for detecting rare and low-abundance mutations	needs to be combined with other detection methods	[42]
		ARMS-PCR	high sensitivity, high accuracy, easy operation, and low cost	unable to achieve high-throughput and high-position detection	[43]
		BEAMing	measurement of individual DNA molecules with high reliability and sensitivity	complex operational procedures	[44]
		NGS	high throughput and high sensitivity	complex data analysis workflow and high cost	[45]
	methylation detection	whole-genome bisulfite sequencing	high sensitivity and coverage of the entire genome	complex operational procedures and high cost	[46]
		DREAMing	simple, cost-effective, and has high sensitivity and specificity	only detects known loci	[47]
		DISMIR	high sensitivity and relatively low detection cost	emphasizes the distribution of methylation across the entire genome rather than individual loci	[48]
**NcRNAs**	detection based on polymerase chain reaction	RT-qPCR, dPCR, and ddPCR	high sensitivity, simple and well-developed analysis program	controversy surrounding the standardization process	[49]
	detection based on next generation sequencing	gene chips and RNA-seq	screening of ncRNAs can be accomplished	reduced specificity and elevated cost	[50]
	detection of expression and function	microarray	no amplification procedures are required	depends on known molecules	[51]
	nanostructured biochips	metallic nanoparticles, graphene oxide, quantum dots, and nanostructured polymers	high sensitivity allows it to detect molecules at very low concentrations	the fabrication process is more complex than traditional biochips	[52]
**EVs**	isolation	ultracentrifugation	ease of operation and low cost	contamination and integrity are compromised	[53]
		filtration	low cost and high efficiency	contamination and not suitable for all body fluids	[54]
		size-exclusion chromatography	high particle integrity and less susceptible to contamination by soluble proteins	low particle yield and not suitable for all body fluids	[55]
		membrane affinity	ease of operation and good commercial viability	low specificity in the enrichment of EVs subpopulations and susceptible to contamination	[56]
		immunoaffinity capture	can separate specific subpopulations of particles	low yield	[57]
		precipitation	high yield with good commercial viability	prone to contamination by soluble proteins	[58]
	detection	traditional EVs detection methods, such as enzyme-linked immunosorbent assay, western blot, flow cytometry, and polymerase chain reaction	low cost	low efficiency and complex steps	[55, 59, 60]
		surface-enhanced Raman spectroscopy	a label-free, high-sensitivity technique	surface dependency	[61]
		electron microscopy	reflect exosome structures	costly and sample processing can potentially modify the inherent properties	[62]
		electro-chemical	high sensitivity and wide measurement range	high dependent	[63]
		surface plasmon resonance	high sensitivity and real-time detection	high cost and surface dependency	[64]
		colorimetric detection	ease of operation	susceptible to external interference	[65]
		alternating current electrokinetic chips	high separation efficiency and sensitivity	complex design and manufacturing, limited to specific applications	[66]

## Biomarkers and detection methods

### Circulating tumour cells

CTCs, which detach from the primary tumour, can enter the circulatory system and travel through the bloodstream. However, the majority of CTCs die in the peripheral blood within 1 to 2.5 hours due to mechanical forces or immune system attacks. Nevertheless, a small fraction of CTCs can survive and initiate distant metastasis [67, 68]. Numerous metastatic precursors within CTCs increase the risk of tumour metastasis and recurrence [69–71]. According to most perspectives, CTCs are believed to exhibit specific differences from primary tumours despite originating from primary tumours. This heterogeneity leads to their detachment from the primary tumour and acquisition of epithelial-mesenchymal transition (EMT) characteristics, facilitating intravascular infiltration and enhancing their potential for metastasis [72, 73].

The analysis process of CTCs mainly involves three stages: enrichment, detection, and characterization. Most enrichment methods are applied based on the surface phenotype or physical properties of CTCs. The CellSearch system, developed using an antibody targeting epithelial cell adhesion molecule (EpCAM), is the sole CTC detection technology approved by the U.S. Food and Drug Administration due to its ability to detect CTCs expressing EpCAM [29, 74]. However, this strategy cannot detect cells with low EpCAM expression due to the potential loss of epithelial antigens during the EMT process [34]. Furthermore, the abundance of CTCs varies across different types of cancer; the CellSearch system is more suitable for tumours with higher CTC abundance [75]. In microfluidic devices, affinity-based separation methods can also be employed. Designing microfluidic devices with varying materials, sizes, and structures to manipulate blood flow patterns creates additional opportunities for interacting CTCs and antibodies [32, 57, 76]. Pahattuge et al. [77] introduced a modular microfluidic system called SMART-Chip. They demonstrated that the SMART-Chip platform could significantly reduce the processing time by more than 50% when handling blood samples obtained from patients with PDAC and colorectal cancer compared to manual sample processing. Furthermore, microfabricated porous membranes can be employed to filtrate and isolate CTCs due to their size, which is larger than that of normal blood cells [78, 79].

CTCs are primarily detected using protein expression, immunocytochemistry, and nucleic acid methods. Flow cytometry allows for the quantitative assessment and characterization of protein expression in CTCs, offering the advantage of evaluating multiple biomarkers to characterize CTCs comprehensively. However, it exhibits lower sensitivity for detecting rare populations of CTCs [80]. Immunohistochemical staining and immunofluorescence are commonly employed techniques for detection and characterization purposes. Immunofluorescence, in particular, enables the visual confirmation of protein expression and localization by fluorescent markers. In the conventional cytofluorimetry approach, isolation is achieved through the utilization of specific antibodies that recognize markers selected on CTCs. This method utilizes monoclonal antibodies specifically targeting antigens expressed by CTCs, which results in the exclusion of CTCs that do not express such antigens but are present in the circulation. Consequently, this presents a challenge in obtaining or developing novel antibodies against specific targets [81]. The flexibility of immunofluorescence technology makes it a powerful tool for studying the protein expression of tumour cells. For instance, with the use of multimarker immunofluorescence panels, researchers can gain a more comprehensive understanding of the distribution and expression patterns of different CTC subtypes [82]. This not only aids in tumour classification and staging but also provides valuable insights for personalized therapy. In addition, CTCs can also be detected using techniques such as high-resolution image scanning, mutational analysis, and single-cell next-generation sequencing (scNGS). The molecular characteristics of CTCs were initially determined on enriched fractions, which provided limited information about tumour heterogeneity. In recent years, the rapidly advancing single-cell sequencing technology has become the preferred method for isolating individual CTCs and studying tumour heterogeneity. These technologies will facilitate the comprehensive characterization of CTCs at multiple omics and functional levels, enabling effective monitoring of the dynamic changes in tumour heterogeneity in individual cancer patients [83, 84].

Although the precise role of CTCs in tumour development remains elusive, they offer a valuable approach for obtaining comprehensive insights into tumours through liquid biopsy. In PC management, CTCs play a significant and beneficial role in patient diagnosis, prognostic evaluation, recurrence monitoring, and treatment decisions. In this regard, we have summarized the clinical applications of CTCs in various aspects of PC management in recent years (Table 2).

**Table 2:** Clinical application of CTCs in PC in recent years

	**Factors**	**Sensitivity**	**Specificity**	**AUC**	**Number of patients**	**Findings**	**Year of report**	**Ref**
**Diagnosis**	CTCs + CA19-9			0.95	80	the combination of CTCs and CA19-9 improves the diagnosis of pancreatic cancer	2022	[85]
	CTCs + Folate receptors + CA19-9	0.978	0.833	0.944	46	folate receptors (+) CTCs, especially when combined with CA19‐9, have the potential as a biomarker for diagnosing pancreatic cancer	2020	[86]
	CTCs + EVs GPC1	1	0.8		22	combining CTCs and GPC1-positive-exosome detection displayed 100% sensitivity and 80% specificity in PDAC	2019	[87]
	CTCs	0.75	0.964	0.867	72	CTCs as a biomarker for diagnosis and staging in pancreatic cancer	2016	[88]
	CTCs + CA19-9	0.67	0.8		52	CA19-9, combined with CTCs analysis, may represent an efficient method for diagnosing pancreatic adenocarcinoma in patients with of solid pancreatic tumours	2017	[89]
**Prognosis**	CTCs + KLF8 + vimentin				40	CTCs expressing Krüppel-like factor 8 and vimentin predict prognosis in pancreatic cancer	2021	[90]
	CTCs			0.8	100	CTCs correctly identified patients with occult metastatic disease preoperatively	2018	[91]
**Recurrence**	CTCs + TIC markers				60	CTCs expressing markers of tumour-initiating cells predict survival and recurrence in PDAC	2017	[92]
	CTCs + vimentin + cytokeratin				50	CTCs expressing vimentin and cytokeratin predict the recurrence of PDAC	2016	[93]
	CTCs				36	the presence or absence of CTCs in the blood of patients with PDAC could help predict the recurrence pattern after surgery	2021	[94]
**Therapy**	CTCs + vimentin + CA19-9				100	significantly reduced CTCs counts were observed after chemotherapy in subjects that responded to treatment	2019	[95]
	CTCs	0.82	0.85	0.871	200	CTCs as a biomarker for 1-year recurrence for chemo-naive PDAC patients	2018	[96]

### Circulating tumour DNA

Cell-free DNA (cfDNA) is a crucial genetic component found in the bloodstream; its origin primarily stems from apoptotic, necrotic, and actively secreted fragments originating from healthy, inflamed, and tumour tissue. These fragments are typically approximately 150–180 base pairs in length [97–99]. CtDNA represents a distinctive subset of cfDNA released into the blood by CTCs. Compared to cfDNA, ctDNA is present in relatively lower amounts in the bloodstream, constituting only 1% (or even less than 0.01%) of cfDNA [98–100]. Most ctDNA fragments have lengths ranging from 160 to 200 base pairs, and they are less influenced by intratumoural heterogeneity compared to tumour tissues [97, 101, 102]. Additionally, ctDNA has a half-life of approximately 15 minutes to 2.5 hours, which means that it serves as a real-time tumour biomarker. In contrast, traditional blood protein biomarkers usually take weeks to manifest, and ctDNA can dynamically reflect the status of a tumour at a specific moment [103, 104]. Furthermore, ctDNA carries tumour-related genomic information, such as gene expression levels, mutations, the methylation status, and microsatellite instability. Compared to traditional biopsy markers, ctDNA is an ideal biomarker, especially for the real-time monitoring of treatment effectiveness and prognosis assessment.

CtDNA detection includes ctDNA preparation, library construction, analysis, and data alignment. One aspect of ctDNA detection focuses on genetic mutations. Single-base mutations have the potential to activate oncogenes, disrupting the balance between oncogenes and tumour suppressor genes, thereby instigating tumorigenesis. Another aspect involves DNA methylation, which plays a role in tumour initiation that is similar to that of DNA mutations [105, 106]. Mutation detection is a vital component of the analysis. Due to the extremely low abundance of ctDNA, employing highly sensitive techniques for detecting tumour mutations is crucial. Conventional approaches rely on polymerase chain reaction (PCR), but recent advancements in PCR and sequencing technologies have paved the way for alternative methods, including quantitative PCR (qPCR), digital PCR (dPCR), droplet digital PCR (ddPCR), and next-generation sequencing (NGS). qPCR allows real-time monitoring of DNA amplification with higher speed, reproducibility, and quantification. NGS platforms offer several advantages, including the ability to screen for unknown mutations and structural and copy-number variations. dPCR and ddPCR involve partitioning DNA samples into thousands or even millions of separate compartments or droplets, effectively reducing background noise associated with traditional methods and enabling the detection of tumour DNA at a variant allele frequency (VAF) below 0.1% [107–109]. In recent years, integrated detection strategies combining gene editing techniques, functional enzymes, and nanomaterials have been developed to effectively increase the net content of mutation fragments, thereby facilitating the identification of target gene mutations within ctDNA [106]. There are various methods for DNA methylation detection. Whole-genome bisulfite sequencing (WGBS-seq) is considered the gold standard for DNA methylation analysis. It can identify partially methylated regions in cancer cells. However, the sensitivity of this method may be compromised by DNA degradation [26, 110].

KRAS mutations are the most prevalent genetic alteration in PC. They are present in over 90% of patients and are considered an early driving factor in PDAC [108]. Castells et al. [111] demonstrated that the presence of KRAS mutations in plasma DNA served as a highly specific molecular marker for diagnosis and prognosis in a PDAC cohort of 44 patients. However, it is essential to emphasize that previous cfDNA sequencing results have not only identified mutations known to exist in tumours but have also uncovered a multitude of variations that are absent in tumour tissues [112]. In particular, some patients undergoing chemotherapy may harbour minimal residual lesions composed of drug-resistant cells. In such cases, the mutations detected in cfDNA in the bloodstream do not exclusively originate from tumour cells. CfDNA may also carry mutations from other sources, including those induced by the disease state or treatment. Undoubtedly, KRAS mutations are one of the vital indicators for evaluating PDAC, and their role has received widespread attention as a primary focus in many PC studies (Table 3). However, overall, the application of ctDNA and mutation analysis in PDAC still requires further strategies to thoroughly assess this detection method.

**Table 3:** Clinical application of cfDNA/ctDNA in PC in recent years

	**Testing methods**	**Markers**	**Number of patients**	**Stages**	**Findings**	**Year of report**	**Ref**
**Diagnosis**	ddPCR	*KRAS*	105	all stages PDAC	ddPCR increased the sensitivity and accuracy of EUS-FNA to 91.6% and 88.6%	2022	[113]
	methylation-specific PCR	*ADAMTS1, BNC1*	39	all stages PDAC	two-gene panel with highly promising sensitivity and specificity for detection of earliest stages of pancreatic cancer	2019	[114]
	methylation-specific PCR	*BMP3, RASSF1A, BNC1, MESTv2, TFPI2, APC, SFRP1 and SFRP2*	95	all stages PDAC	patients with PDAC have a highly significant number of hypermethylated genes compared to patients with benign pancreatic diseases	2016	[115]
**Prognosis**	ddPCR	*KRAS*	108	all stages PDAC	mutant-KRAS detection in the blood of PDAC patients is significantly associated with dismal prognosis for palliative and curative patients	2023	[116]
	NGS	*TP53, KRAS*	145	all stages PDAC	cases that had detectable plasma ctDNA showed significantly shorter recurrence-free survival	2022	[117]
	BEAMing	*KRAS*	61	metastatic PDAC	RAS mutation analysis in cfDNA more accurately predicted the prognosis than tissue analysis	2020	[44]
	ddPCR	*KRAS*	104	metastatic PDAC	patients with metastases and detectable ctDNA had significantly shorter progression-free survival and overall survival times than patients without detectable ctDNA	2019	[118]
**Treatment**	ddPCR	*KRAS*	70	metastatic PDAC	the change in magnitude of ctDNA during systemic treatment allows the prediction of treatment response and is associated with both overall survival and progression-free survival	2022	[119]
	dPCR	*KRAS*	47	metastatic PDAC	KRAS mutation in ctDNA during chemotherapy could be a predictive factor for the disease progression of patients with PDAC	2020	[120]
	PCR-based-SafeSeqS assays	*KRAS*	112	resectable PDAC	a 24% decrease in the proportion of patients with detectable ctDNA following surgery	2019	[121]
	ddPCR	*KRAS*	78	localized, metastatic, and recurrent PDAC	no detection or disappearance of KRAS ctDNA within 6 months of treatment was significantly correlated with therapeutic responses to first-line chemotherapy	2019	[41]

### Noncoding RNAs

NcRNAs were once perceived to have a limited impact on tumour initiation and progression due to their inability to encode proteins. However, emerging evidence has highlighted the essential regulatory functions of ncRNAs. In addition to their capacity to modulate gene and protein expression, ncRNAs actively participate in diverse tumorigenic processes, including EMT, autophagy, and apoptosis [122–124]. NcRNAs can be classified into two main categories based on their lengths: small noncoding RNAs (sncRNAs), with a length of less than 200 nucleotides, and long noncoding RNAs (lncRNAs), with a length exceeding 200 nucleotides [125, 126]. In addition, circRNAs, which are RNA molecules with a circular structure, have been recognized for their significant regulatory roles in gene expression, cell proliferation, cell differentiation, and disease development in recent years. SncRNAs encompass several subtypes, including microRNAs (miRNAs), small nucleolar RNAs, small nuclear RNAs, piwi-interacting RNAs, and tRNA-derived small RNAs [127]. Among them, miRNAs are the most extensively studied factors in cancer research, and liquid biopsy identifies miRNAs actively secreted by CTCs and tumour cells themselves [128, 129]. MiRNAs can influence genes, with thousands of miRNAs regulating approximately 60% of the genes. Their principal function involves binding to recognition sites in the 3' untranslated region, thereby reducing mRNA stability and suppressing gene expression [130, 131].

LncRNAs play a regulatory role in protein and miRNA functions and expression levels and contribute to chromatin remodelling [130, 132]. Some lncRNAs are considered valuable biomarkers for PDAC because they mediate various processes involved in tumour cell progression, making them applicable for liquid biopsy in PDAC [122]. The detection methods for ncRNAs are similar to those for cfDNA and ctDNA. Standard techniques include qPCR, dPCR, ddPCR, gene chips, and NGS. Over the past few decades, many miRNA detection methods have been developed. The most commonly used methods include qPCR, hybridization chain reaction, rolling circle amplification, strand displacement amplification, and the use of media, such as graphene oxide and gold nanoparticles, to transfer probes into cells [133–136]. Zhao et al. [137] established a novel miRNA and circRNA detection approach based on an enhanced fluorescent signal. This method exhibits a significantly improved detection sensitivity and can be applied to both miRNA and circRNA detection. Additionally, Dittmar et al. [138] successfully applied the Abcam Fireplex™ (a novel limited-volume assay platform) to identify differential plasma miRNAs between early-stage PC cases and controls. Based on hydrogel particles, this platform enables the detection of up to 68 miRNAs in 20 μL of plasma per sample in a 96-well plate format without extensive isolation and purification steps. Likewise, improved detection methods can save lncRNA extraction and purification steps. Lou et al. [139] proposed a rapid colorimetric method to detect lncRNA HOTTIP in diluted serum; one significant benefit of this assay is its ability to be performed on diluted serum, eliminating the requirement for RNA extraction and purification. This detection method holds tremendous potential in PDAC clinical screening. In conclusion, these new technologies significantly differ from traditional detection methods. Although further advancements are required for their clinical application and efficacy validation, these technologies have opened up new avenues for detecting ncRNAs.

### Extracellular vesicles

EVs are essential heterogeneous subcellular structures involved in intercellular communication and are composed of a phospholipid bilayer membrane with membrane proteins and glycoproteins [140]. EVs contain many bioactive molecules, including mRNAs, miRNAs, nucleic acids, lipids, proteins, transcription factors, and growth factors [140–142]. Viable cells actively secrete EVs and exhibit a ubiquitous presence in various bodily fluids. These vesicles are pivotal in enabling essential cellular communication within specific pathological and physiological contexts. Remarkably, tumour cells are also capable of releasing EVs, which actively participate in various mechanisms encompassing the initiation and progression of cancer, immune regulation, and neuronal communication [143]. According to the position statement released by the International Society for Extracellular Vesicles in 2018 [144], vesicles with a size of < 100 nm or < 200 nm are referred to as "small EVs," while those larger than 200 nm are termed "medium/large EVs." Based on their size and cellular origin, EVs can be classified into three main subtypes: exosomes, microvesicles, and apoptotic bodies. Exosomes have received considerable attention and have been extensively investigated among these subtypes. Initially considered cellular debris, similar to ncRNAs, exosomes have emerged as important molecules due to their involvement in diverse biological processes, including molecular transport, intercellular communication, and immune responses [145, 146]. The size of exosomes falls within the range of 30–160 nm [145, 147], classifying them as small EVs. Exosomes present distinctive advantages in the field of liquid biopsy. Compared to ctDNA released from apoptotic or necrotic cells, exosomes released by viable cells provide a more representative depiction of active tumour cell information. Additionally, exosomes exhibit better stability than ctDNA, which is attributed to their protective lipid bilayer [148–150]. With ongoing technological advancements and improved analytical capabilities, exosomes have the potential to become one of the most important alternative tools for liquid biopsy, and we have summarized the clinical applications of exosomal cargo or ncRNAs in various aspects of PC in recent years (Table 4).

**Table 4:** Clinical application of exosomal cargo or ncRNAs in PC in recent years

	**Exosomal cargo or ncRNAs**	**Type**	**Source**	**Number of patients**	**Findings**	**Year of report**	**Ref**
**Diagnosis**	miR-1246 and miR-4644	miRNA	saliva	12	the relative expression ratios of miR-1246 and miR-4644 were significantly higher in the cancer group than these ratios in the control group	2016	[159]
	miR-191, miR-21 and miR-451a	miRNA	serum	61	miR-191, -21, and -451a enclosed in serum exosomes, significantly up-regulated in pancreatic cancer, were sensitive diagnostic markers	2018	[160]
	miR-155	miRNA	pancreatic juice	35	ex-miR-21 and ex-miR-155 levels discriminated PDAC patients from chronic pancreatitis patients with area under the curve values of 0.90 and 0.89, respectively	2019	[161]
	glypican-1	protein	serum	190	ROC curves indicated that GPC1 + circulating exosomes revealed a perfect classifier with an AUC of 1.0, a sensitivity of 100%, and a specificity of 100%	2015	[102]
**Therapy**	chitinase 3-like-1 and fibronectin	protein	macrophage	N/A	CHI3L1 and FN1 induce resistance of PDAC cells to GEM, and inhibitor treatment has proven effective	2021	[162]
	miR-20a-5p	miRNA	plasma	73	the relative expression of miR-20a-5p in gemcitabine-resistant plasma of PC patients was significantly lower than in nonresistant patients	2019	[163]
**Recurrence**	miR-451a	miRNA	plasma	56	miR-451a showed the highest upregulation in the stage II patients who showed recurrence after surgery	2018	[164]
	miR-4525, miR-451a and miR-21	miRNA	portal vein blood	55	high expression of miR-4525, miR-451a, and miR-21 in portal vein blood was associated with recurrence with higher sensitivity and specificity than that in peripheral blood	2019	[165]
**Prognosis**	miR-3607-3p	miRNA	NK cells	40	miR-3607-3p is down-regulated in pancreatic cancer and decreased miR-3607-3p level predicts poor prognosis in pancreatic cancer patients	2019	[166]
	IARS	circRNA	peripheral blood	79	circRNA IARS is highly expressed in pancreatic cancer, positively correlated with tumour metastasis, and negatively correlated with patient survival time	2018	[167]
	UPK1A-AS1	lncRNA	serum	75	the patients with higher UPK1A-AS1 expression had a shorter PFS than those with lower UPK1A-AS1 expression	2022	[168]

The isolation and characterization of EVs present certain challenges. One challenge arises from the low abundance of EVs in biological samples, necessitating highly sensitive techniques for their isolation and purification. Moreover, EVs are prone to contamination from non-EV proteins, lipoproteins, and high-density lipoproteins [151, 152]. Currently, major methods for EV isolation include the following: density-based (ultracentrifugation), size-based (filtration and size-exclusion chromatography), affinity-based (membrane affinity and immunoaffinity capture), precipitation (polyethylene glycol precipitation), and microfluidic technologies. Each method for extracellular vesicle isolation has advantages and limitations. The choice of method should be based on a comprehensive consideration of factors such as the intended purpose, sample characteristics, and experimental conditions. For example, ultracentrifugation, considered the gold standard for EV isolation, is time-consuming and may lead to potential damage to EVs [152]. Filtration is a simple and convenient method. However, it presents challenges in effectively removing impurities from the filter membrane and can still result in the deformation and lysis of EVs [153]. Novel techniques based on microfluidics have been developed to isolate EVs; size-based microfluidics use nanowire and micropillar structures to separate EVs with diameters in a certain range from smaller cellular debris, proteins, and other particles [154, 155]. Compared to conventional approaches, the consolidation of EV isolation and disease detection on a unified platform enhances the clinical viability of EV detection. Microfluidic technology offers significant advantages in this regard, as it allows for preserving EV morphology while minimizing contamination from proteins and other nanoparticles. Additionally, microfluidics exhibits characteristics such as portability, rapidity, low cost, and ease of operation, which are crucial for noninvasive disease detection [152, 155]. Zheng et al. [156] developed an alternating drop-shaped micropillar array to assist in capturing tumour-derived exosomes by Tim4-modified magnetic beads to improve the efficiency of exosome capture. This approach enables the effective extraction of tumour-derived exosomes and greatly enhances their purity. Combined analysis of different types of biomarkers on exosomal membrane surface proteins improves the accuracy of cancer diagnosis. Emerging EV isolation techniques have allowed EVs to rival other important biomarkers, such as CTCs and ctDNA. However, further refinement is still required to optimize this technology for practical clinical applications [154].

Once EVs are extracted, they need to be measured and examined. Taking exosomes as an example, common analysis methods for detecting specific proteins include western blot, enzyme-linked immunosorbent assay, and flow cytometry techniques. However, these methods often require expensive equipment or relatively long analysis times [61, 157, 158]. Currently, there have been numerous reports on other EV detection and characterization methods, such as electrochemical, colorimetric detection surface-enhanced Raman spectroscopy (SERS), surface plasmon resonance (SPR), and biosensors based on nanomaterials [61, 63–65]. Li et al. [61] developed a direct and sensitive strategy for detecting exosomes in serum samples using hierarchical SERS substrate and detection probes. The superposition of hotspots between the hierarchical SERS substrate and SERS probes, combined with the proximity of SERS probes achieved through magnetic bead aggregation, resulted in a dual enhancement of the Raman signal. This not only enhances the sensitivity of exosome detection but also holds significant potential for early PC diagnosis. EVs have demonstrated tremendous potential and promising diagnostic, prognostic, and therapeutic prospects. The emergence of these novel detection methods and tools has contributed to the early diagnosis and therapeutic assessment of PC. However, it will take time to integrate them into clinical workflows and disease management strategies. While continuous technological innovation and improving isolation yields are crucial, clinical translation should be given equal attention.

## Clinical applications in the management of PC

### Early diagnosis

Currently, the main diagnostic methods for PC are EUS-FNA, MRI, and CT. However, most patients do not experience symptoms until the tumour obstructs the bile duct or invades surrounding nerves [34, 169]. Nonetheless, early detection is possible. Yachida et al. [170] proposed a diagnostic window of at least 10 years from the initial formation of a pancreatic tumour to the onset of symptoms, which provides a critical opportunity for early detection of PC through liquid biopsy. Early diagnosis can improve the success and survival rates of treatment and effectively reduce the side effects and cost of treatment.

CTCs are detectable at all stages of PDAC, including the precancerous lesion stage. However, CTCs can detach from the primary tumour and infiltrate the bloodstream, a crucial pathway for metastasis in advanced stages. Consequently, CTCs are seldom present in the early stages of the tumour, and detecting PDAC in the precancerous and early stages is challenging due to the limited sensitivity of CTC detection techniques [29]. These limitations currently impede the implementation of CTC-based early detection and screening of PDAC in the population. Nonetheless, the high specificity of CTCs renders them a valuable auxiliary diagnostic tool, as they are almost undetectable in healthy individuals [108]. A study conducted by Ankeny et al. [88] encompassed a cohort of nearly half of the early-stage PDAC patients (43.1% early-stage I/II), and CTCs demonstrated a sensitivity of 75.0% and specificity of 96.4% in the diagnosis of PDAC, with significant differences in CTC counts between PDAC and nonadenocarcinoma diseases. Compared to CTCs, which are often detectable in the late stages of cancer, circulating epithelial cells (CECs) may have the potential to identify patients earlier in the disease process because haematogenous dissemination may occur before tumour formation [171]. Rhim et al. [172] developed a sensitive method for labelling and tracking pancreatic epithelial cells in a mouse model of PC and detected pancreatic-origin CECs in the precancerous stage. After two years, the research team conducted a prospective blinded trial [173], employing a previously utilized detection platform for CTCs in patients with prostate cancer. The results indicated that among the 19 control individuals, only three individuals (15.8%) exhibited detectable CECs, with a maximum count of 3 CECs/ml. Conversely, in the cohort of 9 PDAC patients, 77.8% (7 individuals) exhibited detectable CECs, while among the 20 patients with pancreatic cystic lesions, 40% (8 individuals) demonstrated detectable CECs. Several other clinical studies have also confirmed the detectability of CECs in patients with benign, precancerous, and malignant pancreatic lesions, especially in those with highly atypical precancerous lesions [174, 175]. The application of single CECs or CECs combined with other molecular markers has demonstrated value in various types of tumours [176, 177]. Additionally, pancreatic-origin CECs may play a crucial role in tumour metastasis and provide initial evidence for the diagnostic value in PDAC, albeit requiring further validation with larger patient cohorts.

Similarly, ctDNA is derived from apoptotic and necrotic tumour cells, which are characteristic of advanced disease [178, 179]. KRAS mutations are commonly used as the primary target in ctDNA analysis. However, a study has demonstrated that single ctDNA analysis for KRAS mutations in plasma samples exhibits poor sensitivity, accuracy, and area under the curve (AUC) (35.2%, 51.0%, and 0.683, respectively) [113]. However, despite the identification of specific mutations in PC, these mutations can be shared with other cells and do not align exclusively with tumour cells [112]. This implies that in the analysis, these mutations cannot be regarded as the sole indicators exclusively associated with the tumour. Therefore, it is necessary to approach the presence and significance of these mutations in a more comprehensive and cautious manner. To provide a more comprehensive assessment of the value of ctDNA in liquid biopsy, many researchers are exploring strategies to combine ctDNA with other biomarkers. Cohen et al. [180] conducted a case‒control study involving 221 surgically resectable PC patients and 182 healthy individuals, and this study integrated the analysis of ctDNA mutations with protein markers. The key contribution of this study is the demonstration that genetic alterations can be detected with elevated protein markers. Importantly, the combination of ctDNA and protein markers performed better than any individual marker in screening tests. In another study [89] focusing on 68 patients with solid pancreatic tumours (58 malignant, 10 benign), a combination analysis involving CA19-9, ctDNA, and CTCs achieved a sensitivity of 78% and specificity of 91% for the diagnosis of PC. Furthermore, a registered clinical study in the United States (NCT03334708) is currently underway, with an anticipated enrolment of 700 participants. This study aims to develop blood-based biomarkers, including ctDNAs, for the early diagnosis and assessment of treatment response in PC. In addition to analysing mutational characteristics in PC, recent research has focused on integrating methylation markers as potential indicators for early diagnosis. For example, the methylation status of ADAMTS1 and BNC1 in cfDNA has demonstrated excellent diagnostic performance in detecting early-stage PDAC (sensitivity: 97.4%, specificity: 91.6%, AUC: 0.95) [114]. Although further optimization may be required for practical clinical applications, methylation markers offer a promising and noninvasive diagnostic strategy for identifying PDAC.

Despite the considerable interest in miRNAs as potential biomarkers, their widespread utilization for the clinical diagnosis of PC is currently lacking. Among the commonly investigated miRNAs, miR-196a, miR-196b, miR-885-5p, miR-122-5p, miR-210, and miR-21 have been extensively studied in this context. However, their application as validated diagnostic markers in routine clinical practice has yet to be established [181–186]. Dittmar et al. [138] employed an innovative hydrogel particle-based miRNA assay platform to analyse fluid samples with limited volume. They successfully identified molecular biomarkers (miR-34a-5p, miR-130a-3p, and miR-222-3p) that demonstrate suitability for stage II PDAC cases. Notably, when combined with CA19-9, the integration of these biomarkers resulted in an increased AUC from 0.89 (using CA19-9 alone) to 0.92, 0.94, and 0.92, respectively. It is important to emphasize the significant value of individual miRNAs in PC diagnosis, as they exhibit a favourable balance between effectiveness and cost-effectiveness. Some diagnostic models incorporating many biomarkers exhibit high diagnostic performance, but their complex composition and low reproducibility present challenges for practical clinical application. A comprehensive review of the literature conducted by Wnuk et al. [187] investigated the clinical value of circulating plasma miRNAs in PDAC. The analysis of 55 circulating miRNAs revealed that 66.10% exhibited superior diagnostic value compared to CA19-9, whereas only 23.73% of miRNAs performed worse. In all cases where miRNAs exhibited inferior diagnostic value compared to CA19-9, combinatorial strategies effectively enhanced the diagnostic performance of these miRNAs. The current reports on lncRNAs and circRNAs as early diagnostic markers for PDAC are limited. This is primarily due to the untapped potential of these molecules as PDAC biomarkers, particularly in the case of circRNAs, which is still in its early stages of research [188]. Most studies on lncRNAs and circRNAs have predominantly focused on their roles as therapeutic targets and prognostic markers for PDAC [189–192]. These studies have provided insights into the involvement of lncRNAs and circRNAs in the initiation and progression of PDAC, suggesting their potential as future diagnostic markers. However, further advancements in bioinformatics methods and functional characterization techniques are needed to fully explore their diagnostic capabilities.

Given the abundant biological information carried by EVs, the molecular contents of these vesicles can mirror crucial phenotypic traits of their parent cells. Consequently, there has been a recent surge in interest among researchers to investigate the potential utility of EVs as biomarkers for early-stage diagnostic purposes [193]. In a study conducted by Yu et al. [194], a comprehensive case‒control analysis was carried out, encompassing a cohort of 501 participants, among whom 284 were diagnosed with PDAC. This investigation aimed to assess the differential extracellular vesicle long RNA (exLR) levels in PDAC patients, individuals with chronic pancreatitis, and healthy control individuals. They developed a robust signature comprising eight distinct exLR molecules that demonstrate exceptional accuracy in discerning stage I/II cancer cases. The merged AUC of the signature reached an impressive value of 0.949. Glypican-1 (GPC1), a cell surface heparan sulfate proteoglycan, is specifically detected on PDAC cell-derived exosomes and not detectable in nontumor cells. Even without MRI results and noticeable pancreatic lesions, mutant KRAS mRNAs can be detected in circulating GPC1 + exosomes in the serum. This implies that GPC1 + exosomes may serve as biomarkers for detecting premalignant lesions [102, 195]. It is crucial to emphasize that the full-fledged implementation of extracellular vesicles in early diagnosis necessitates the standardization of their isolation and detection techniques, alongside the conduction of more extensive clinical investigations. Presently, an ongoing clinical study in the United States (NCT05625529) is focused on liquid biopsy and the utilization of extracellular vesicles for the early detection of PC.

### Monitoring treatment response and resistance

Although surgery is the preferred treatment for PC patients, less than 20% of patients diagnosed with the disease can undergo surgical treatment due to the limitations of early detection methods [196]. Gemcitabine (GEM) is commonly used as a first-line treatment for PC, particularly in advanced-stage patients, and is considered highly effective. However, chemoresistance is a common issue that significantly limits the effectiveness of this treatment. Developing personalized treatment strategies with the help of liquid biopsy technologies before treatment is one potential solution to address this problem.

There is still some controversy regarding whether CTCs can predict treatment response. Most studies have found that a decrease in CTC counts signifies a favourable treatment response [73, 95, 96]. However, some research suggests that there may not be a significant difference in CTC counts between blood samples collected before and after chemotherapy, possibly due to variations in CTC identification and treatment strategies [29, 93, 197]. In addition to quantitative analyses, the molecular characteristics of CTCs are also frequently used to assess a patient's treatment response [198]. Some CTC measurement techniques enable genetic profiling of CTCs, allowing the detection of key gene mutations, such as those in KRAS, HER2, and TP53 [199–201]. Furthermore, programmed death ligand 1 (PD-L1) staining methods can be employed to evaluate the status of CTCs in patients receiving monoclonal antibody therapy, with PD-L1-negative CTC patients often achieving better treatment outcomes [202]. In most cases, CTCs express chemokine receptors, with CXC-motif chemokine receptor 4 (CXCR4) being the most commonly expressed receptor. Continuous monitoring of CXCR4 during treatment serves as a predictive biomarker, providing information to identify which patients are likely to benefit from treatment or develop resistance [203, 204]. Regarding drug sensitivity, Wu et al. [205] conducted a study wherein they collected CTCs from patients diagnosed with PDAC and expanded them ex vivo into organoids. The sensitivity of these organoids to nine drugs (GEM, 5-fluorouracil, erlotinib, irinotecan, olaparib, oxaliplatin, paclitaxel, palbociclib, and trametinib) was examined. A significant correlation was observed between the drug sensitivity of CTCs and clinical outcomes. This indicates that the drug sensitivity of CTCs holds the potential to predict therapeutic outcomes in PDAC, thus enabling the avoidance of ineffective treatments. CECs have been proposed as a potential tool to predict how patients will respond to antiangiogenic cancer therapies. However, it is important to recognize that their diverse phenotypes may exhibit different dynamics during the course of treatment. Given the unique characteristics of CECs and their crucial role in liquid biopsies, this avenue of research holds promise and warrants further exploration. A clinical trial focused on late-stage pancreatic cancer patients monitored CEC levels during neoadjuvant therapy and observed an overall increase in CECs in response to combination therapy that was attributed to chemotherapy-induced vascular damage exacerbating CEC release [206]. Furthermore, research concerning surgery, which is a common treatment method, has indicated that CEC levels typically decrease after tumour resection. This decline may result from the disruption of PDAC-derived growth factor recruitment of endothelial cells after tumour removal, subsequently reducing CEC levels [207].

The longitudinal assessment of ctDNA enables dynamic monitoring of disease trajectory, including treatment monitoring and the detection of minimal residual disease, and serves as an alternative biomarker for overall disease burden [208]. Tao et al. [209] conducted a study to examine the role of ctDNA in monitoring treatment response in a cohort of 17 PDAC patients who were treated with the FOLFIRINOX regimen (fluorouracil, irinotecan, and oxaliplatin). Among the 12 patients who responded to chemotherapy, 11 exhibited a reduction in the mutant allele fraction (MAF) of cfDNA. In contrast, the remaining 5 patients who developed chemotherapy resistance showed an increase in the ctDNA MAF during disease progression. These findings suggest that the levels of ctDNA partly reflect the tumour burden. In another study, Groot et al. [210] identified a substantial decrease in the probability of detecting ctDNA in the bloodstream of patients who underwent neoadjuvant chemotherapy compared to those who did not receive any preoperative chemotherapy (21% vs. 69%; *p* < 0.001). Although the practice of longitudinal ctDNA monitoring in PDAC cases remains limited, these studies underscore the potential of ctDNA as a crucial monitoring biomarker during the therapeutic course.

The emergence of chemoresistance presents formidable obstacles for nonsurgical candidates, thereby exacerbating their clinical predicament. Several miRNAs are considered key regulatory elements involved in acquiring chemoresistance in PDAC. Lu et al. [163] demonstrated that the expression of plasma miR-20a-5p in PDAC patients who exhibited resistance to GEM was markedly diminished compared to that in nonresistant patients (*p* < 0.01). The authors proposed that miR-20a-5p potentially regulates the expression of the RRM2 protein, thereby exerting an influence on the sensitivity of tumour cells to GEM. MiRNA levels have the potential to serve as informative indicators regarding disease progression, whether assessed before treatment initiation or during the treatment course. In a study by van der Sijde et al. [211], the elevated expression levels of serum miR-373-3p before FOLFIRINOX therapy was identified as a predictive factor for disease progression. Correspondingly, the reduced expression levels of miR-194-5p following a single cycle of FOLFIRINOX treatment indicated disease deterioration. LncRNA holds considerable importance in guiding the therapeutic approach for PC. Zhang et al. [168] demonstrated that UPK1A-AS1 expression significantly facilitates chemoresistance to oxaliplatin in PDAC. Elevated UPK1A-AS1 expression is directly associated with an unfavourable chemotherapy response and shorter progression-free survival in patients with advanced PDAC. These findings underscore the potential of ncRNA as a valuable tool for monitoring treatment response and evaluating tumour drug resistance in PDAC.

EVs play a pivotal role in acquiring GEM resistance in PDAC, particularly under conditions of prolonged drug exposure. Mikamori et al. [212] elucidated that extended GEM treatment upregulates the expression of miR-155 within PDAC cells. Moreover, they have substantiated a positive correlation between miR-155 expression levels and the secretion of extracellular vesicles, which promote GEM resistance in clinical samples. Nevertheless, it is imperative to acknowledge that the development of GEM resistance in organisms is not invariably irreversible. In a separate investigation [162], the authors unveiled the capability of macrophage-derived extracellular vesicular factors, namely, chitinase 3-like-1 (CHI3L1) and fibronectin (FN1), to induce resistance to GEM in PDAC cells. Treatment interventions involving the administration of CHI3L1 and FN1 inhibitors, canertinib and pifithrin, respectively, have demonstrated partial restoration of GEM resistance. These findings suggest the potential utilization of these inhibitors as adjunctive therapeutic modalities in managing GEM-resistant PDAC patients.

### Evaluation of prognosis, metastasis, and recurrence

Accurate prognostic assessment is crucial for determining appropriate treatment strategies in patients with surgically resectable PC. It should be emphasized that ideal biomarkers are generally considered to possess both high sensitivity and high specificity. However, very few biomarkers can perfectly exhibit both high sensitivity and high specificity, even within excellent multimarker panels [213]. It is typically necessary to find a reasonable balance between these two aspects to meet varying clinical needs and objectives. In diagnosis, diagnostic tests with high specificity are typically used to determine whether a patient has a specific disease. However, highly sensitive circulating biomarkers are more useful in prognosis assessment, as they can detect the ongoing progression or recurrence of a disease, enabling health care professionals to implement timely therapeutic measures.

CTCs and ctDNA also play a significant role in prognosis assessment, providing information that can reveal the tendency for tumour metastasis and recurrence, as well as the overall survival (OS) of PC patients. This viewpoint has been confirmed by several studies [91, 94, 214–217]. Although the CTC count is commonly used as the determining criterion in studies, distinguishing CTC subgroups can also reflect the tumour status to some extent. Certain CTC subgroups with specific phenotypes, for example, indicate the tendency of the tumour for metastasis [218, 219]. Semaan et al. [33] identified and characterized 4 CTC subpopulations that can be used for the clinical stratification of PC, providing a valuable perspective for applying liquid biopsy technologies in prognostic prediction. Some studies suggest that CTC distribution shows spatial heterogeneity and that portal venous blood may be a better option for assessing PDAC prognosis than peripheral venous blood [220]. The CTCs count from intraoperative portal venous blood has been linked to poor prognosis in resectable PDAC patients [221]. Some studies have focused on the factors expressed on the surface of CTCs. In a study of 55 PDAC patients, Nitschke et al. [222] discovered that CTC positivity (≥ 3 CTCs) was significantly linked to shorter recurrence-free survival (*p* = 0.002). Moreover, they proposed the expression of RARRES1 on CTCs as a novel biomarker for treatment failure and early recurrence. During follow-up observations, some PDAC patients experience tumour recurrence after surgery. Interestingly, as the tumour recurs, the number of CECs increases once again. This phenomenon may be linked to the ongoing release of CECs by recurrent tumours, and these cells may play a role in tumour growth and invasion [207]. Many studies suggest that pretreatment levels of CECs are not associated with treatment response, specific disease stages, histological adverse prognostic features, or overall survival [174, 223–225]. However, monitoring specific subsets of CECs with overlapping phenotypes reveals a unique potential for prognosis prediction in PDAC, which may help in more accurately predicting disease progression and survival for patients [206].

Research on ctDNA primarily focuses on three KRAS mutations (G12D, G12V, and G12R) [226]; however, G12R has a lower detection rate than the other two mutations [227, 228]. Ako et al. [227] studied postoperative recurrence and overall survival in PDAC by analysing two KRAS mutations (G12D and G12V); they discovered that patients with both KRAS mutations had significantly lower disease-free survival. Interestingly, in a separate study, Guo et al. [216] specifically examined the influence of the G12D mutation on the prognosis of PDAC. Among 26 patients with PDAC, those with the KRAS G12D mutation had notably reduced overall survival (12.1 vs. 24.9 months, *p* < 0.001) and recurrence-free survival (6.3 vs. 17.4 months, *p* < 0.001) compared to those without the mutation. Notably, patients with the KRAS G12D mutation exhibited a distinct early recurrence trend and poorer clinical outcomes.

In previous studies, several reports have confirmed the predictive role of ncRNAs in PC. Most of these studies constructed multifactor prognostic risk models and performed survival analyses with these models [189, 229, 230]. Kandimalla and colleagues [231] developed a risk model for PDAC that includes nine miRNAs (miR-192-5p, 194-5p, 194-3p, 215-5p, 375-3p, 552-3p, and 1251-5p). They used this model to predict the survival outcomes of PDAC patients and evaluated the feasibility of applying the model to liquid biopsy. The results showed that PDAC patients with high-risk scores had a significantly shorter 5-year OS rate than those with low-risk scores (8.6% vs. 48.4%; HR = 2.85 [95% CI 1.41–5.76]; *p* = 0.02). This study helps identify high-risk patients and predict the prognosis of PDAC patients in clinical settings. However, such studies typically need to be validated in larger populations, and an excessive number of factors in the model hinder its application in clinical practice.

In terms of EVs, Reese et al. [232] demonstrated the diagnostic and prognostic potential of serum exosomal miR-200 in PDAC in the context of extracellular vesicles; this study showed that low expression of miR-200b in EpCAM-positive serum exosomes and miR-200c in total serum exosomes were favourable for the prognosis of PDAC. Clinical studies have shown that various substances can be detected in purified exosomes from the plasma of tumour patients. Tumour cells overexpressing PD-L1 can evade immune system surveillance and invade neighbouring tissues. A study [233] found that patients with systemic advanced PDAC had high levels of exosomal PD-L1. Nevertheless, the statistical analysis did not show a significant correlation between the level of exosomal PD-L1 and survival outcomes. In addition to the PD-L1/PD-1 axis, other immune checkpoints have also been found to be involved with exosomes. The CD155/TIGIT axis, for instance, plays a role in tumour immune evasion, similar to the PD-1/PD-L1 axis. High expression of CD155 and TIGIT has been associated with adverse prognosis in many tumours [234–236]. Furthermore, PDAC cells have been shown to express high levels of survivin. Exosomes containing survivin can induce tumour resistance and provide an additional advantage for tumour progression. Research has indicated that KRAS-dependent cancer cells produce exosomes rich in survivin, promoting cancer cell survival and resistance and ultimately leading to poor prognosis [237]. Some studies have attempted to integrate different biomarkers, which seem to have greater advantages. Yang et al. [238] discovered that combining multiple factors offers more advantages than using a single biomarker. They developed a five-factor biomarker panel for blood samples that includes EV-CK18 mRNA, EV-CD63 mRNA, EV-miR-409, cfDNA concentrations, and CA19-9. This biomarker panel exhibited not only excellent diagnostic capabilities for PDAC (with an accuracy of 92%, sensitivity of 88%, specificity of 95%, and AUC of 0.95) but also outperformed traditional imaging methods in detecting the occult metastasis of PDAC (with an accuracy of 84%, sensitivity of 78%, specificity of 88%, and AUC of 0.85).

## Limitations and prospects

In the face of the threat posed by PC, surgery appears to be the only curative method, but only for patients without detected metastasis. However, due to the limitations of early diagnosis techniques, 80% of patients miss the opportunity for surgery and resort to chemotherapy. Nevertheless, chemoresistance remains an unavoidable problem. Even with surgery, some patients may still experience tumour metastasis during the operation or shortly after surgery, as precise prognosis evaluation is not always possible. Currently, liquid biopsy in clinical practice aims to address the following three issues: 1) achieving an early and accurate diagnosis of PC to provide patients with more treatment options; 2) accurately assessing metastasis and recurrence risks before surgery to avoid unnecessary treatments; and 3) selecting appropriate chemotherapy drugs and accurately evaluating treatment response for patients who are not eligible for surgical resection.

**Fig. 2:**
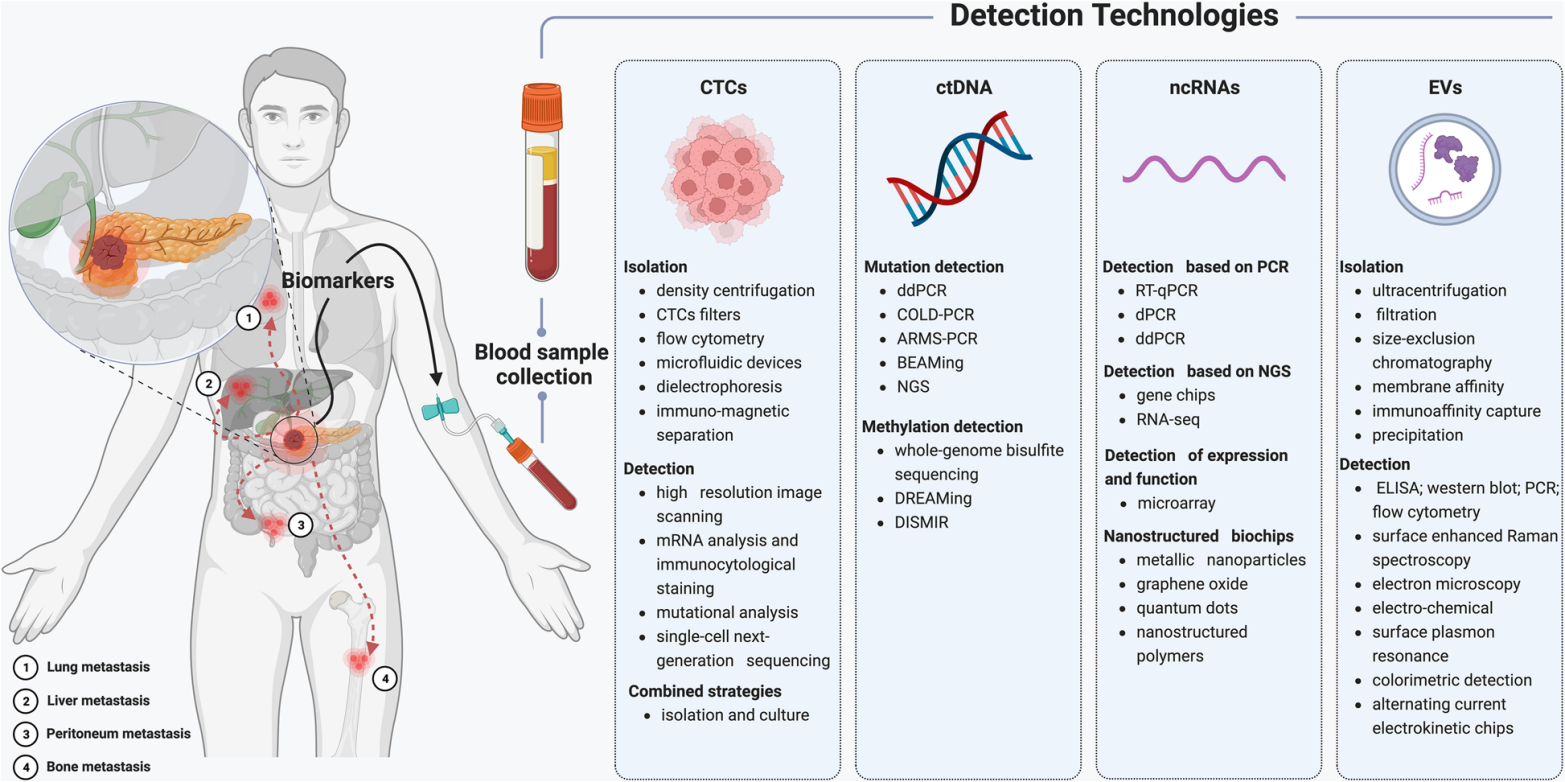
Common metastasis pathways of pancreatic cancer and detection techniques of liquid biopsy. Tumour cells, molecules, and biomarkers originating from the tumour itself are considered significant factors in the metastasis of pancreatic cancer. The liver is the most frequent site of metastasis in pancreatic cancer, followed by the lungs and peritoneum. Currently, various detection techniques are employed to identify different biomarkers, aiming to acquire more tumour characteristics. Created with BioRender.com

In recent years, liquid biopsy has shown promising prospects in disease diagnosis, treatment monitoring, and prognosis assessment [239–241]. As a noninvasive detection method, liquid biopsy is more advantageous than traditional tissue biopsy in terms of both economic and convenience aspects, especially when tissue samples cannot be obtained. Although cells, molecules, and biomarkers originating from tumours can potentially promote tumour metastasis, we can leverage detection technologies in this process to enhance the precision of disease diagnosis (Fig. 2). Disease progression is dynamic, highlighting the need for liquid biopsy to enable multiple sampling and dynamic observation of disease development. Based on liquid biopsy results, physicians can make more informed clinical decisions and improve disease treatment strategies, which is particularly urgent for diseases with high mortality rates, such as PC.

We should acknowledge that liquid biopsy still has certain limitations. First, liquid biopsy is still in its developmental stage with relatively low technological maturity; the complex detection process and inefficient equipment hinder its wider adoption. For example, the low concentrations of CTCs and ctDNA lead to reduced sensitivity, while contamination during sample extraction and processing may result in false-positive and false-negative outcomes. Different sample processing methods also result in low sensitivity and specificity of liquid biopsy; therefore, improving the detection level is the key to further developing and applying liquid biopsy. Second, liquid biopsy is a new detection technology that lacks standardized operating procedures and uniform data analysis methods, which can affect its accuracy and reliability to some extent. It is necessary to develop standardized analytical processes alongside technological improvements to enhance its clinical application value. Additionally, the occurrence and development of diseases involve the interaction of multiple organs, cells, and various biological molecules. Thus, an isolated liquid biopsy can only reflect the level of specific molecules or biomarkers and cannot comprehensively reflect the complex features of diseases. Therefore, it is necessary to consider integrating other examination indicators, such as CT, MRI, and ultrasound. Finally, most research results cannot be directly applied in clinical practice and require rigorous prospective trials in larger populations. Strict validation and evaluation should be conducted before clinical application. In summary, we believe that the future of liquid biopsy development should prioritize the development of new technologies and analysis platforms while simultaneously improving the operating and analytical procedures. In addition, large-scale clinical trials should be actively promoted.

## Conclusion

In conclusion, the application of liquid biopsy in the clinical management of PC aligns with the concept of precision medicine. Biological samples obtained through noninvasive procedures can provide detailed information about various aspects of the tumour, which aids in monitoring tumour development and evaluating treatment responses. Moreover, this information assists clinical physicians in understanding the molecular mechanisms of tumour occurrence and development and providing more accurate and personalized treatment decisions for each patient. There are also some limitations, including low-sensitivity detection techniques, nonstandardized analysis workflows, and small sample sizes; these limitations are significant barriers of liquid biopsy. It cannot be denied that with the continuous advancement of technological methods and large-scale clinical trials, many biomarkers have begun to demonstrate their value, indicating broad prospects for their application. Liquid biopsy will become an indispensable technology for tumour diagnosis and treatment.

## Data Availability

Not applicable.

## Abbreviations

PC: Pancreatic cancer
PDAC: Pancreatic ductal adenocarcinoma
EUS-FNA: Endoscopic ultrasonography-guided fine needle aspiration
MRI: Magnetic resonance imaging
CT: Computed tomography
CA19-9: Carbohydrate antigen 19-9
CTCs: Circulating tumour cells
ctDNA: Circulating tumour DNA
ncRNAs: Noncoding RNAs
mRNAs: Messenger RNAs
EVs: Extracellular vesicles
EMT: Epithelial-mesenchymal transition
EpCAM: Epithelial cell adhesion molecule
scNGS: Single-cell next-generation sequencing
cfDNA: Cell-free DNA
PCR: Polymerase chain reaction
qPCR: Quantitative PCR
dPCR: Digital PCR
ddPCR: Droplet digital PCR
NGS: Next-generation sequencing
VAF: Variant allele frequency
WGBS-seq: Whole-genome bisulfite sequencing
sncRNAs: Small noncoding RNAs
lncRNAs: Long noncoding RNAs
miRNAs: MicroRNAs
SERS: Surface-enhanced Raman spectroscopy
SPR: Surface plasmon resonance
CECs: Circulating epithelial cells
AUC: Area under the curve
exLR: Extracellular vesicle long RNA
GPC1: Glypican-1
GEM: Gemcitabine
PD-L1: Programmed death ligand 1
CXCR4: CXC-motif chemokine receptor 4
MAF: Mutant allele fraction
CHI3L1: Chitinase 3-like-1
FN1: Fibronectin
OS: Overall survival
